# Covert therapeutic micro-processes in non-recovered eating disorders with childhood trauma: an interpersonal process recall study

**DOI:** 10.1186/s40337-022-00566-1

**Published:** 2022-03-21

**Authors:** Malin E. Olofsson, KariAnne R. Vrabel, Asle Hoffart, Hanne W. Oddli

**Affiliations:** 1 Department of Psychology, University of Oslo, Forskningsveien 3A, 0373 Oslo, Norway; 2 Research Institute, Modum Bad, Vikersund, Norway

**Keywords:** Eating disorders, Trauma, Process research, Working alliance, Qualitative methods

## Abstract

**Method:**

To uncover therapeutic micro-processes from the perspectives of eating disorder (ED) treatment non-responders with childhood trauma (CT) late effects, we explored in-session experiences of poor long-term outcome patients. Female inpatients aged 28–59 (*M* = 40.2, *SD* = 5.0) from a randomised trial comparing Compassion Focused Therapy for EDs (n = 3) with Cognitive Behavioural Therapy for EDs (n = 3) were interviewed with video-assisted recall about a self-selected session. Data were analysed through Interpretative Phenomenological Analysis (IPA) with Grounded Theory (GT) elements.

**Results:**

Covert patient strategies included self-effacement, regulating therapeutic distance to open up, and engaging with reflective rather than experiential interventions. First, self-effacement included submissive, passive or pretend responses to perceived criticising or violating therapist behaviours as well as other orientation and submission for approval. Second, some preferred a close patient–therapist alliance with therapist self-disclosure and reciprocity was a requirement for opening up; others required distance. Third, informants detached from experiential trauma work while engaging in joint reflection on post-trauma responses.

**Conclusion:**

Informants were preoccupied with calibrating the emotional–relational landscape in session; we hypothesized that psychological insecurity and affective intolerance from CT limit their freedom to explore own in-session experiences.

**Supplementary Information:**

The online version contains supplementary material available at 10.1186/s40337-022-00566-1.

## Plain English summary

A patient perspective on individual ED treatment processes is scarce for difficult-to-treat eating disorders (EDs) with childhood trauma (CT). We therefore interviewed six poor long-term outcome inpatients through video-assisted recall about a self-selected therapy session. Patients’ covert in-session strategies included self-effacing behaviours in relation to their therapist, their respective preferences for closeness or distance to their therapist to be able to open up in session, and being more prone to engage in therapists’ reflective interventions rather than experiential. In sum, patients were preoccupied with calibrating the emotional–relational landscape between patient and therapist in session, which were strategies that went undetected. We hypothesized that a lack of psychological security and affective tolerance limit patients freedom to explore own experiences from the perspective of traumatic attachment and self-differentiation theory.

**Supplementary Information:**

The online version contains supplementary material available at 10.1186/s40337-022-00566-1.

## Background

Childhood trauma (CT) seems more common in eating disorders (EDs) than in the general population: prevalence rates range from 37 to 100% depending on CT definitions [1]. Compared to EDs without CT, the ED-CT link is associated with earlier ED onset, greater bingeing/purging, dissociation, suicidality, psychiatric comorbidity, and ED severity, alongside an increased probability for treatment drop-out [2–7]. ED treatments are lacking for this patient group [8, 9], and directions for integrating ED and trauma treatments do not exist [1].

CT is a risk factor for ED development, and trauma symptoms may reinforce the ED as ED behaviours may facilitate avoidance of distressing trauma symptoms, and thus decrease hyperarousal [10]. A network analysis of ED and PTSD bridge symptoms found associations between bingeing and irritability, body dissatisfaction and distressing dreams, and shape difficulties and concentration difficulties [11]. CT in EDs also associate with avoidant coping and ED symptoms in a dose–response manner [12], supporting that childhood sexual abuse with avoidant personality predict ED chronicity [13]. In fact, avoidance, rumination and suppression of emotional expression are all linked to increased ED severity. Little is, however, known about how ED patients modify their emotions in relation to *others* [14], which is relevant as therapists’ and patients’ moment-to-moment regulation strategies seem to interact [15].

Patients themselves similarly connected EDs with CT to emotional, interpersonal and self-image regulation difficulties in two patient-as-expert studies. Bingeing was perceived as a strategy for comfort and relieving trauma-related emotions, and purging following bingeing for weight control to avoid rejection and abandonment. Bingeing without purging was conversely perceived as protection from sexual abuse through weight gain [16, 17]. Our study added the repeated use of ED behaviours to build mastery and cure helplessness/emptiness, and the ED was conceptualised as a secure base and emotion regulation strategy in the absence of co-regulation abilities [17]. CT late effects with EDs thus seem pervasive beyond PTSD, at least for some, following complex PTSD (CPTSD; [18]).

Foreseeably, interpersonal problems and attachment insecurity may affect the ability to engage in productive working alliances [19], which both parties contribute to through constant negotiation of mutual goals, task collaboration, and emotional bond [20]. However, while the alliance quality has repeatedly proven to be a reliable predictor of moderate size for treatment outcome across disorders and orientations [21, 22], its role in ED treatment has been unclear [23, 24]. A meta-analysis, however, concluded that the alliance-outcome association was partially accounted for by early symptom reduction, as early ED symptom improvement predicted subsequent alliance quality and vice versa. The alliance’s impact on outcome also varied on a subgroup level, being stronger for younger patients (cf. older patients) with AN (cf. other EDs) and weaker for treatments with a strong behavioural component [25].

Quantification of the alliance is nevertheless problematic, as there may be qualitative differences to the constructs measured [22]. Notably, patients’ views as opposed to independent raters’ or therapists’ views hold strongest clinical validity [25] and little is known about the treatment processes that concur with non-responses or iatrogenic effects. Reflecting openly on unfavourable outcomes is relevant since understanding treatment failures may guide treatment modifications [26, 27]. Thus, patient perspective inquiries exploring features of *un*productive alliances as well as productive ones merit attention.

Our patient-as-expert process-outcome study broached aspects of emotion and alliance experiencing as we compared change processes in inpatient ED treatment for good (n = 4) versus poor (n = 7) long-term outcomes. Salient processes for good outcomes were: fostering patient agency, engaging in trauma exposure and creating a balance between self-assertion and vulnerability within the therapist-patient dyad. Central processes in poor outcome patients were: orientation towards others’ needs, distrust in their own abilities and difficulties showing vulnerability, in addition to not addressing trauma and maintaining either a distanced or idealised therapist relationship [28]. Nevertheless, patients’ moment-to-moment experiences remain undisclosed, calling for research on treatment processes in relation to outcomes [29].

One strategy to disentangle such fine-grained processes is through Interpersonal Process Recall (IPR; [30]), which facilitates the retrieval of unspoken experiences through video-assisted recall. An early IPR study revealed outpatients’ (diagnoses unknown) general tendency to act deferentially towards their therapist (fear of criticising the therapist, eagerness to meet therapist expectations, and concern about the approach), and responses depending on what they feel they can disclose safely and whether they feel comfortable approaching inner experiences [31]. Another IPR study revealed that outpatients’ (diagnoses unknown) emotional disengagement (withdrawing, distancing, or lessening intensity) may be both productive and obstructive, e.g., protecting oneself from potentially painful content, choosing what to disclose to maintain safety, testing therapist responses to avoidance and vulnerability, and interrupting self-impeding patterns [32]. Yet another study revealed that patients with anxiety, depression, PTSD, and personality disorders experienced inner struggles when attempting to open up during the initial phases of affect-focused therapy, for example fearing the intensity and consequences of negative emotions, feeling disloyal to loved ones, insecurity about one’s right to share inner experiences, and being bodily stuck and unable to open up [33]. We believe that video-assisted recall is yet to be used to study treatment micro-processes in EDs and CT late effects.

Consequently, we aim to extend previous research by uncovering moment-to-moment processes in patients with EDs and CT late effects through the research question: How do ED treatment non-responders with CT late effects experience in-session processes within the context of the patient-therapist working alliance? Thus, by identifying covert processes from the patients’ vantage point, we aim to pinpoint therapeutic processes in poor long-term ED outcomes within the framework of an inpatient randomised controlled trial (RCT).

## Method

### Setting and overarching RCT

The study was conducted in a tertiary care psychiatric hospital in southeast Norway, treating various disorders. The hospital is part of the public health care system and admits patients on a nationwide basis free of charge. Our sample comprised of patients from a highly specialised eating disorder unit, and specifically those participating in an RCT comparing 13-week inpatient Compassion-Focused Therapy for EDs (CFT-E) with Cognitive-Behavioural Therapy for EDs (CBT-ED) in EDs with or without CT (Clinical trials: NCT02649114) with the assumption that CFT-E would be more beneficial for EDs with CT. The trial protocol is described in detail elsewhere [34]: the RCT findings are yet to be concluded. Our study targeted micro-processes within the patient-therapist dyad for poor long-term ED outcome inpatients with psychiatric sequelae from emotional, physical, and/or sexual CT.

### Treatments

#### CBT-ED

CBT-ED [35] is the first choice ED treatment targeting cognitive-behavioural change and maintaining factors, e.g., disordered eating, compensatory beliefs about needing to control weight/food intake, and core beliefs about low self-worth. CBT-ED incorporates traditional CBT techniques such as graded exposure and cognitive restructuring alongside ED-specific targets such as dietary change. If late CT effects play a role in ED maintenance, imaginal exposure, and rescripting is incorporated. For more information on CBT-ED within the RCT, see trial protocol [34].

#### CFT-E

CFT-E [36] focuses on emotion regulation and shame and self-criticism as ED maintenance factors, which frequently occur in EDs with CT and also seem involved in the maintenance of post-traumatic sequelae [37, 38]. CFT-E aims to balance patients’ threats, soothing, and drive systems, and promote compassion for self and others. This is achieved through mindfulness and visualisation techniques and by giving and receiving compassionate support in groups. See trial protocol [34] for the dissemination of CFT in the current setting.

### Diagnostic assessment

Experienced clinicians from a separate assessment team conducted MINI and SCID-II, and two psychology students blinded to group allocation and trained in diagnostic assessment conducted EDE-16. Therapists and participants were not blinded to group allocation, since the treatments in the RCT were of a psychological nature.

#### General psychopathology

The MINI International Neuropsychiatric Interview (MINI; [39]) is a diagnostic interview that assesses DSM-IV axis I disorders in adults, such as psychosis and depression. The MINI has good psychometric properties across treatment contexts [40, 41].

#### Personality disorders

The Structural Clinical Interview for DSM-IV axis II disorders (SCID-II; [42]) was used to assess personality traits. SCID-II includes 13 DSM-IV personality disorders. Patients were assessed using the standard categorical approach: they qualified for a personality disorder if they fulfilled a predetermined number of criteria.

#### Eating disorder

The Eating Disorder Examination Interview 16.0 (EDE-16; [43]) is a 42-item semi-structured interview that guided clinicians’ assessment of ED pathology during the previous four weeks (Restraint, Eating concern, Shape concern, Weight concern), summarised in a Global EDE score. Most items are rated on a 7-point Likert scale (0–6), except for the frequency of ED behaviours. The Norwegian EDE-16 has acceptable internal consistency and inter-rater reliability [44].

### Self-report measures

#### Eating pathology

Eating Disorder Examination Questionnaire 6.0 (EDE-Q; [45]) is a 28-item self-report questionnaire measuring the frequency and intensity of ED symptoms during the previous four weeks (e.g., “a definite fear of losing control over eating”). Symptoms are rated on a 7-point Likert scale, where a global score indicating ED severity is divided into four subscales: Restraint, Eating concern, Shape concern, and Weight concern. The Norwegian EDE-Q has shown acceptable internal consistency, reliability, and validity [46]. Cronbach’s alpha for our sample was 0.89 at assessment.

#### Childhood trauma

A 28-item short form of the Childhood Trauma Questionnaire (CTQ-SF; [47]) guided patient inclusion in the RCT (completed at assessment). CTQ-SF measures the severity of sexual, physical, and emotional abuse alongside physical and emotional neglect. Each subscale has five items graded on a 5-point Likert scale *(1* = *never true, 5* = *very often true)*. CTQ has satisfactory psychometric properties across Norwegian samples [48]. Cronbach’s alpha for the total scale in our sample was 0.79.

#### Trauma symptoms

PTSD Symptom Scale Self-report version (PSS-SR; [49]) measures seventeen DSM-IV PTSD symptoms during the previous week, rated on a 4-point Likert scale *(0* = *not at all, 3* = *always or five or more times weekly)*. A total score measures PTSD severity divided into three subscales assessing trauma-related hyperarousal, re-experiencing, and avoidance. The Norwegian PSS-SR has good concurrent validity and test–retest reliability [50]. Cronbach’s alpha for our sample at assessment was 0.92.

#### Interpersonal problems

The 64-item version of the Inventory of Interpersonal Problems (IIP-64; [51]) measured problematic interpersonal behaviours on a 5-point Likert scale (0 = *not at all, 4* = *extremely)*: 39 items measuring difficulties regarding others (*It is hard for me to…*) and 25 items concerning behaviours that patients feel they are doing too much regarding others (*Things that you do too much*). The subscales correspond to octants in a circumplex model: cold, vindictive, domineering, intrusive, self-sacrificing, overly accommodating, non-assertive, and socially inhibited. There are two dimensions to the model: agency (y-axis) and communion (x-axis). Norm scores are calculated to determine patients’ caseness for each variable [54], meaning that a total score exceeding 1 represents caseness. The psychometric properties of the Norwegian IIP-64 are acceptable [52], and Cronbach’s alpha for our sample at assessment was 0.85.

#### Therapeutic working alliance

The 12-item short form of the Working Alliance Inventory (WAI-SR; [53]) measures the patient-therapist quality during the previous week rated on a 7-point Likert scale *(1* = *never, 7* = *always)*. Data were collected weekly during treatment starting the third week, and the scale was divided into three subscales based on Bordin’s [20] working alliance theory: agreeing on treatment goals (Goals), agreeing on tasks to achieve those goals (Tasks), and the quality of the patient-therapist relationship (Bond). The 3-factor model has demonstrated good psychometric properties, internal consistency, and convergent validity across countries and contexts [54, 55]. Cronbach’s alpha for our sample ranged from 0.75 to 0.93.

### Case selection

All RCT participants from March 2016 to February 2017 who fulfilled the inclusion criteria of an ED diagnosis (DSM-5; [56]) with CT (CTQ) were considered eligible. To define accurate case determination for the RCT, the recommended CTQ scoring options by Walker and colleagues [57] was used. Threshold scores for each of the five subscales were based on receiver operating characteristic methods, with very good to excellent sensitivity and specificity (≥ 0.85) for each of the five subscales. Hence, patients scoring ≥ 8 on the sexual abuse, physical abuse, or physical neglect subscale or ≥ 10 on the emotional abuse subscale or ≥ 15 on the emotional neglect subscale were considered trauma patients in the RCT, thus eligible for participation in the study. Exclusion criteria were current psychosis, ongoing trauma (e.g., still being in an abusive relationship), or suicidality requiring extensive care, i.e., acute suicidality not manageable in settings that function on voluntary admissions.

A poor long-term outcome was defined as not meeting either full or partial recovery at 1-year follow-up, defining full recovery as (a) not fulfilling an ED diagnosis, (b) no restraint, bingeing, and purging for 3 months, (c) BMI > 18.5, and (d) EDE-Q subscale scores within 1 *SD* of age-matched community norms. For partial recovery (d), psychological ED components remained [58]. Criteria a and b were assessed through EDE-Q scores and clinicians’ opinions, and Norwegian EDE-Q norms were compared against Australian age-matched norms to ensure correspondence [59, 60]. Accordingly, 18 RCT inpatients were eligible for the current study during this period. Two declined participation, and four were not introduced to the study for unknown reasons, resulting in 12 possible informants. Technical challenges and poor video sound quality then ruled out five informants, narrowing the sample down to seven possible informants who had recordings of acceptable quality. Of these, six had poor outcomes at the 1-year follow-up according to the aforementioned outcome criteria, and were chosen for analysis (Table 1), meaning that data saturation was not involved in determining sample size, but rather, informants were selected for analysis post-interview.

**Table 1:** Case selection of non-recovery based on assessment at 1-year follow-up

Alias	ED diagnosis	ED behaviour	BMI	Restraint	Eating concern	Shape concern	Weight concern	EDE-Q total	Outcome category*
Elizabeth	Yes	Yes	45.97	**6**.**00**	**4**.**40**	**5**.**63**	**4**.**60**	**5**.**16**	Poor
Sandra	Yes	No	24.17	**3**.**00**	**1**.**80**	**5**.**63**	**5**.**20**	**3**.**91**	Poor
Helena	Yes	Yes	21.97	0.00	**2**.**40**	2.25	1.60	1.56	Poor
Martha	Yes	Yes	37.29	**4**.**80**	**6**.**00**	**5**.**25**	**4**.**40**	**5**.**11**	Poor
Alice	No	Yes	19.00	**3**.**60**	0.20	0.90	1.60	1.60	Poor
Maria	Yes	Yes	23.57	**5**.**00**	**3**.**60**	**6**.**00**	**6**.**00**	**5**.**15**	Poor

Values exceeding Eating Disorder Examination Questionnaire (EDE-Q) cut-offs in bold

*Based on recovery criteria [76] and Norwegian norms [46]

### Participants

#### Informants

Six white Norwegian females with EDs and CT and an average age of 40.2 years (*SD* = 9.8, range 28–59) were interviewed post-treatment (three CBT-ED, three CFT-E). Five (83%) had experienced at least three trauma types, most commonly emotional neglect (n = 6, 100%), followed by emotional abuse (n = 5, 83%), physical neglect (n = 5, 83%), physical abuse (n = 3, 50%), and sexual abuse (n = 2, 35%). Average ED duration was 28.2 years (*SD* = 6.4, range 14–54), with 12 years as the mean age for ED onset (*SD* = 1.6, range 6–17). Patients had on average been in treatment for 11.2 years prior to admission (*SD* = 4.0, range 1–26) (Table 2).

**Table 2:** Patient pre-treatment characteristics and treatment in the RCT

Alias (age group)	Diagnoses (DSM-5)	Trauma (CTQ)	ED duration (years)	Treatment duration (years)	Treatment in trial	Therapist Treat Exp. (years)	Session no
Elizabeth (45–50)	BN	SA, PA	40	26	CBT-ED	8	8/26
	PTSD	PA, EN					
		EA					
Sandra (25–30)	OSFED (subthr. AN)	EA, EN	14	3	CFT-E	8	22/26
	MDD	PN					
	Avoidant PD						
	Borderline PD						
Helena (40–45)	BN	EA, EN	29	5	CFT-E	4*	5/26
	PTSD	PN					
Martha (55–60)	BED	PA, PN	53	1	CBT-ED	1	20/26
		EN, EA					
Alice (25–30)	OSFED (subthr. AN)	PA, PN	14	16	CFT-E	4*	5/26
	PTSD	EN, EA					
	MDD						
Maria (35–40)	OSFED (subthr. AN)	SA, EN	19	16	CBT-ED	8	18/26
	Avoidant PD						

CTQ, Childhood Trauma Questionnaire [47]; Treatment duration = total years in psychiatric in- or outpatient treatment, TreatExp., therapists work experience particularly with EDs; BN, bulimia nervosa; PTSD, post-traumatic stress disorder; DDNOS, dissociative disorder not otherwise specified; OSFED, other specified feeding and eating disorders, Subthr. AN, subthreshold anorexia nervosa; MDD, major depressive disorder; PD, personality disorder; SUD, substance use disorder; SA, sexual abuse; PA, physical abuse; PN, physical neglect; EN, emotional neglect; EA, emotional abuse

*The same therapist working with two patients

#### Therapists

Five white female therapists (four clinical psychologists, one psychiatrist) specialised in ED treatment for 5.8 years on average participated (Table 2). They were trained in the respective treatments and received regular supervision from the primary treatment developers as part of the RCT. Neither therapists nor supervisors were part of the research team.

#### Researchers

Our team comprised white Norwegian-speaking psychologists with clinical/research experience: a female clinical psychologist, a female associate professor with ED expertise, a male professor in clinical psychology, and a female associate professor in clinical psychology. We have competence in CBT, CFT, existential therapy, systemic and narrative/language-based, and affect/emotion-focused models.

### Procedure and data collection

A semi-structured interview guide was developed according to Interpersonal Process Recall (IPR; [30]), where informants were prompted through open-ended, exploratory questions (Additional file 1). IPR is an audio-or video-assisted recall method that aims to facilitate the retrieval of “conscious yet unspoken experiences” [61, p. 18], in this case, covert treatment processes. The methodology is a joint process of meaning-making as the interviewer and informant together attempt to make the implicit explicit through dialogue. This allows for reflexive co-analysis beyond informants’ actual experiences there and then while removing unnecessary constraints to discovery [62].

Individual therapy sessions took place twice weekly as part of the 13-week inpatient treatment; they were recorded and stored on a local server at the treatment facility for fidelity purposes in the RCT. Informants were briefed about the research project by their therapists as they approached discharge, and the first author informed those interested in the project and asked for informed written consent. The first author viewed and summarised all available video recordings prior to each interview and briefly presented their content to the informants at the start of each interview. Next, informants were instructed to select one session that they considered important regarding experiencing therapeutic work and collaboration processes, as best as they could consider the time interval between session and interview. The IPR interview was originally designed to be conducted within 48 h of the video-recorded session, but has been practiced at longer intervals [63, 64]. In our study, time intervals were compromised for practicality reasons and took place on one occasion, on average, 170 days post-discharge (*SD* = 56, range 11–339). Due to the evocative nature of the IPR method, in which informants are exposed to their previous sessions through the videos, these time intervals were not considered to threaten the validity of informants’ recalls of their own experiences [65].

Interview data was collected between November 2016 and February 2017, meaning that interviews took place before case selection of poor outcome cases at 1-year follow-up. The six selected videos occurred between Sessions 5 and 22 out of 26 sessions over 13 weeks (two weekly, Table 2). The first author replayed the selected video on a 13″ screen laptop computer to stimulate recall and shared exploration, and the informants were instructed to pause the video whenever they could recall any significant experiences that they wanted to explore further. Four interviews were conducted during post-treatment in informants’ homes, one at the treatment facility during 1-year follow-up, and one at the University of Oslo, due to informants’ wishes.

### Data analysis

Interviews lasting between 97 and 140 min were audiotaped, and both video-recorded sessions and interviews were transcribed verbatim, creating parallel conversations. Interpretative Phenomenological Analysis (IPA; [66]) with theoretical roots in phenomenology, hermeneutics, and idiography was deemed especially suitable since our data was rich and complex, and concerned the personal experiences of a small sample. In practice, IPA researchers should try to put aside their taken-for-granted assumptions about the world to the extent possible to capture the phenomenon under study (bracketing) and focus on making sense and interpreting informants’ sense-making of their life worlds (double hermeneutics) while protecting the nuances of each informant’s narrative (idiography). In IPA, the individual has precedence over the general, and accordingly, themes were first identified in a case-to-case manner to capture individual nuances. Master Themes (MTs) resulted from the further analysis that aimed to capture commonalities across the sample, and therefore represent abstractions of individual themes as opposed to themes that were salient across the whole sample. We added the “coding for process” element from Corbin & Strauss’s [67] version of Grounded Theory (GT), meaning that we explored patterns and variation in patient-therapist interactions across dyads, or “how the main issues /…/ are handled through action and interaction by participants, and how change in response relate to changes in condition” [67, p. 293].

All analyses were conducted in Norwegian. The first author read through the transcripts while listening to recordings to immerse in data. Transcripts were then analysed separately in a case-to-case manner focusing on processes, and the first author discussed emerging themes with the fourth author. Specifically, each subsequent interview was sensitised by previous interviews, meaning that we sought to label themes following previously labelled themes. The second and third authors then read through the transcripts to become familiar with the data, and they were presented with interpretive higher-order themes by the first author, which were concluded conjointly as deviations were addressed. Finally, quotes were translated by the first author with a double master’s degree in English and Nordic languages.

### Trustworthiness

The trustworthiness of the study was addressed [68]. Subjectivity and researcher bias were managed by making assumptions overt through ongoing reflexive discussions, which was necessary since two members of our team had central roles in the RCT. The fourth author with the most experience in qualitative research audited the process, and we used purposeful sampling to identify good exemplars of the phenomenon under study and multiple data sources (interview, self-report, video recordings) to obtain thick descriptions. Also, informants had unlimited time to express themselves, and four were interviewed in their homes, which enhanced the interpretive status of the evidence. Data immersion from data collection to communication of results helped to achieve a deeper understanding of the data and ensured the groundedness of the themes.

### Ethical considerations

It was explicitly communicated verbally and in writing that participation was voluntary, that participants were free to decline at any time without giving a reason, and that non-participation would not influence possibilities for future treatment. Due to the presumed vulnerability of the patient group in combination with sensitive material in the videotapes (e.g., trauma exposure and viewing ones’ body) that could potentially evoke difficult emotions or heighten the risk of self-destructive behaviours, the interviewer was available for contact via phone and e-mail before and after the interviews. We established that informants had therapists locally at the time of the interview to ensure their safety. All procedures were approved by the Norwegian Regional Committee for Medical Research Ethics (nr 2015/2160) and conducted according to the Helsinki declaration.

## Results

**Fig. 1:**
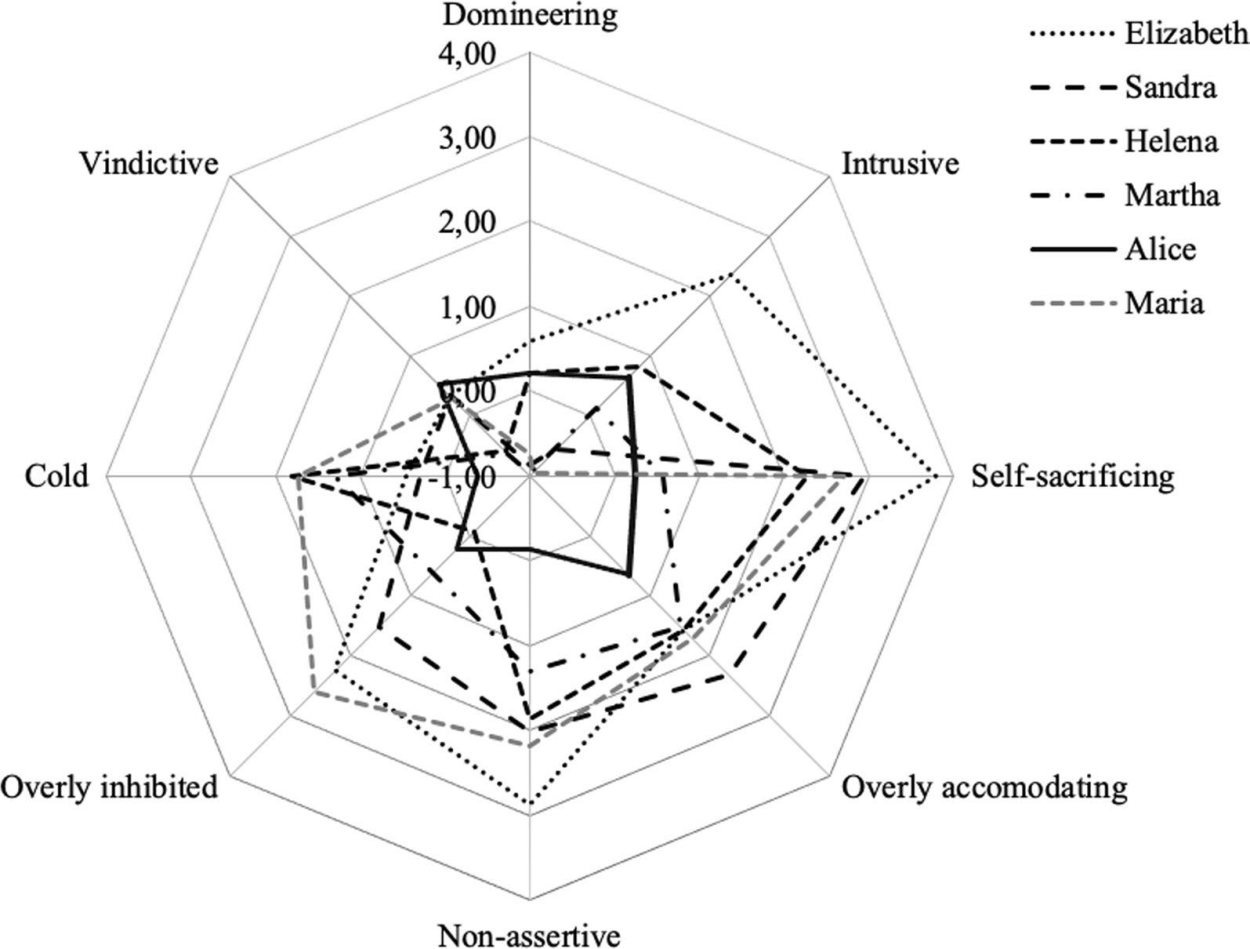
Individual IIP-64 scores at the start of treatment

All informants scored within the clinical range on EDE-Q (*M* = 4.5; *SD* = 0.7, range 3.5–5.1) pre-treatment. Four (all except Alice and Maria) also scored within the clinical range PTSD range *(M* = 20.5; *SD* = 11.0, range 6–30). IIP-64 tendencies leaned towards the non-agentic pole with mixed scores for the communion dimension (Fig. 1), except for one outlier (Alice) who did not score for caseness on either PTSD or interpersonal problems. WAI weekly subscale scores ranged from 1 to 7 (Additional file 2: Fig. S1, Additional file 3: Fig. S2, Additional file 4: Fig. S3). The transcriptions rendered 47 excerpts relevant to the study’s aim; in 30 of the cases, the informant paused the recording.

Informants expressed interest in the video; however, several mentioned initial reflective difficulties due to being visually exposed to their weight/shape, and overall, they needed prompting to work their way into the IPR method and the video. The overarching themes self-effacement, regulating closeness, and distance to the therapist to be able to open up and emotional avoidance during trauma work across five informants are illustrated below, followed by the narrative of a diverging case (Alice), who described agency and emotional experiencing while supported by the therapist. All themes are exemplified in Table 3, in which patients’ accounts are displayed regarding each therapy segment.

**Table 3:** Example excerpts

Therapy segment	Informant account
Master Theme I: P managing interactions with T through self-effacing and submissive behaviours (Elizabeth, Sandra, Helena)	
Theme A «P feeling rebuked by T; P non-assertive to avoid verbal abuse; P becoming submissive» (Elizabeth)	
Context: T and P exploring a conflict between P and a fellow patient during a group therapy session	P: I feel that I’m being rebuked by the therapist. I feel rebuked, and I really hate that. /…/ It ruins my trust in her. Rebukes are in a way an insult to my intelligence, right? Because I do understand that it’s childish, but… things get really big [in treatment], right? Things that you would (blows) back home. /…/ Well, I’m really uhm (.) straightforward as a person, but I’m also scared to step on other peoples’ toes (.). And maybe… when I was a little girl, if I protested, I got hit, right? And I feel that… nobody has hit me (.) my husband hit me once, and that’s the biggest insult I’ve ever experienced, and I actually threatened to kill him. And being rebuked to me, it’s like being punched in the face. That’s why I always get insecure about whether or not to speak my opinion, because I’m scared of, not being physically violated but getting hit verbally. (Elizabeth)
T: But what if you could try to turn things around? What you’re saying right now is really good; that you want to be there for others and so forth, but what if you could turn things around a bit and think that you’re all adults? Of course, it’s important that those things are addressed, that you say them out in the open. And I really think that you’re addressing important topics [in group therapy]. And she heard all of what was said. Uhm, and whether she decides to consider it or not, I think that’s up to her …and something that may have to be worked on with *her* individually…	
P: (overlapping) Yes	
P: For sure. I haven't felt that	
P: Mm	
Theme B «P fearing to fail therapy and upset T; P other-orienting to find ‘correct’ answers» (Sandra)	
Context: T and P exploring P’s emotions in relation to a social media incident	P: I think it works because when she asks questions, I think about them for quite a long time. Because I don’t know what to answer- I *know* what to answer, but I don’t dare saying. And then it turns into that I start thinking about how I can express myself correctly so that it doesn’t come out wrong. And there it is, the constant fear of saying something wrong. Across all relationships, really. /…/ I talked to a friend just now, like you said, my thoughts are like «What am I supposed to answer that’s correct?». (Laughing). That’s what’s so exhausting, in life generally. That I’m always like «Am I saying something wrong now?», «Am I doing something wrong now?», «Are you angry with me?», «You are, you are, you are». (Sandra)
P: I was just about to throw up. I get physically ill	
T: Because of what happened?	
P: Mm	
T: That’s a pretty strong reaction to something, so…	
P: Yeah, it’s goes rather deep	
T: Yes, how do you feel about that? Do you, do you understand why it’s so deeply rooted?	
P: (shaking head)	
T: Any thoughts… or ideas?	
P: I’m afraid to make mistakes	
T: What happened to you in the past when you made mistakes?	
P: (.) (inaudible) punished for it	
T: Yes. Things that weren’t your fault? Right?	
P: But I can’t get myself to think like that… I really can’t help it. (laughing)	
Theme C «P feeling violated by T; P contempting T but pretending to participate; P increased self-contempt» (Helena)	
Context: T and P discussing whether or not P’s total exercise time weekly exceeds what is allowed in treatment	P: There you go! Scornful! Like «get your act together». it’s all over my face, «seriously?» (sighing). Can you see the gaze I’m giving her? bit contemptuous. Like «Seriously. Come on. What are we doing here?». I’m not pleased here; I can see that. But I participate, and play along, but I’m not at all happy about it. /…/ It comes out as self-contempt, you know, since I’m a liar for not saying anything about it. Instead it comes out as «I’m completely stupid, and I’m really sick» and… Yeah. (Helena)
P: Yes, and I lift weights for about 45 min, then perhaps *30 min* of weights, and 30 min cardio	
T: (interrupting) You lost me there, one more time?	
P: I do 45 min of cardio	
T: You just said half an hour	
P: Yes, but I exercise more than once a week	
T: Yes, each time?	
P: Yes, each time. And I also do 30-min weights and 30 min cardio on one occasion (laughing)	
T: Yes, that’s 2 h. No, that’s 1 h of jogging, half an hour weights, and half an hour cardio, that’s 1 h	
P: That’s 1 h. And then I also do 45 min of weights, during one occasion	
T: Yeah? Then it’s 3 h	
P: Yes, and	
T: (interrupting) Then you have 1 h 15 min left for exercise if you are to use 4 h [weekly]	
P: Yeah, and then I do weights for 1 h	
Theme D «P submitting to T to gain approval» (Elizabeth, Helena)	
Context: T and P disagreeing as to whether compulsive exercise is part of P’s eating pathology	P: Look at that. I’m actually sitting there lying. I *remember* sitting there lying. Because all of a sudden, by the end of that session, I had a compulsive exercise disorder. I almost admit it. But it (stuttering) was pure lying. /…/ No, I had just said that «no, I don’t [find exercise problematic]… I’m really conscious of that», and then I suddenly said «but I can also hear that the eating disorder comes and tells me that I have to exercise». So, I’m really saying «no, but yes…». /…/ Because I’m trying to please her /…/ I want to be a good patient. I want to be good and say what I’m supposed to. (Helena)
P: I really feel that when the bulimia doesn’t stick, the eating disorder comes and tells me that I can also use exercise as a means for losing weight, right?	
Theme E «P attempting to self-assert; P fears hurting T and experiences increased guilt; P withdraws assertive attempts» (Helena)	
Context: T and P discussing whether or not P’s total exercise time during a week exceeds what is allowed in treatment	P: I clearly state that I’m scared to make mistakes right there. And I’m usually not like that. «I actually worsen from your regime». I say that clearly there and then. But then I laugh, right? Because I’m not supposed to criticize her. Because it’s really important for me to say «Look, no offence, but I feel that what you’re doing here is a bit silly». Because normally I’m very relaxed in relation to exercise. /…/ I don’t want to hurt her feelings, right? I don’t want to say to her «Look, your regime is not working, it makes me more disordered, done». But I can’t say that to her because then she will feel incompetent. /…/ I automatically think that she needs to leave the session feeling good. That’s something I have very strongly in me. (Helena)
P: It’s good to jog, or it’s good for my heart. But I hate jogging	
T: For how long do you jog?	
P: (hesitating) Say, 1 h. And I lift weights a lot; I like that	
T: Is that 1 h, too?	
P: No (laughing) Today it was (laughing) (inaudible)	
T: (laughing)	
P: No, but I’m talking about (inaudible) during exercise. We are just exercising legs and upper body (inaudible) just to make use of it, and that’s 30 min, so then I still have 45 min I can use. And then I turn into four and a half [years old]… normally I don’t hurry when I exercise, but now I feel a bit like «I have to reach this» and «I can’t take any breaks»	
Theme F «P experiencing T as too forceful; P becoming overwhelmed and passive» (Elizabeth)	
Context: T and P discussing the aftermath of a previous trauma exposure session	P: Perhaps I feel she’s missing out on the fact that (.) that I’m not really interested, because so much is being said *to* me, you know? /…/ Yeah, I feel that I’m not active enough in treatment, really. I liked her, but I was very much *told* things, right? And that might suit some people, I think, that it’s totally fine to some, but when I look at it retrospectively, I feel that I need to be more actively involved in treatment /…/ I feel that I’m being talked t*o*, to a large degree. And I don’t get responses to what *I’m* saying. (Elisabeth)
T: What you’re talking about now is really interesting, Elizabeth. Definitely. You mentioned that you experienced a horrible nightmare. You said you felt persecuted after our latest session. How have you been since, in general?	
P: Well, I’ve felt much more depressed than usual	
T: To the extent that you cannot endure it?	
P: Yeah. You can drag me through anything, and I’ll endure it in a way, but	
T: (interrupting) But did you expect to get a stronger reaction than you actually did?	
P: Yes	
T: Yes. So, you’re really a bit surprised that you didn’t	
P: (interrupting) Yeah. To be able to bring it out and look at it. I think that the thing you did where I split into two—the little one and the grown-up—it helped a bit	
T: Perhaps you experienced a bit more control?	
P: Yeah	
T: And also, the fact that you’ve now become a grown-up	
Master Theme II: P preferring either closeness or distance to T to be able to open up (Elizabeth, Sandra, Martha, Maria)	
Theme G «P distancing from T to be able to open up about affect-laden, trauma-related topics» (Maria)	
Context: T and P addressing P’s dissociative experiences	P: I feel that I was met in a very good manner, uhm. I was being met with understanding, I guess. /…/ yeah, well, she has a lot of expression in her face, uhm, you might not see it /…/ And that’s one thing. But I guess it has to do with what she’s like as a person, too /…/ Yeah, it’s really, it’s like a therapist-patient relationship in a way /…/ And I’ve learnt that, when it’s like that I can be more open. /…/ Yeah, because, uhm, it’s become a learnt situation, I have so much more difficult being open to other people. /…/ It’s in a way a person that I don’t have to adhere to in my daily life either. (Maria)
P: I wrote down a little bit about how (fetching notebook) afterwards how (looking in notebook), uhm, no, it was, I became, I really became very down (inaudible), pushed from a cliff in a way, getting a lot of depressive thoughts, that’s really what it is, thoughts I’ve been having (inaudible), a painful, all-consuming experience took all of me, so I feel (laughing) that I become so damn	
T: (interrupting) To think about it in a way, that’s right, yes	
P: Uhm, not really, uhm when I experienced it, I felt that I really wasn’t’ present (reaching paper towels to wipe tears)	
T: Oh	
P: Yes (laughing while crying)	
T: And that activates something, the experience that you’re not really *there*	
P: Yes	
Theme H «T self-disclosing; P feeling closeness and blurred professional boundaries; P more interested and opening up» (Elizabeth, Sandra)	
Context: T and P explore the topic of self-care	P: There it is, you see that I become more interested, because now, she tells me about herself /…/ and «my job is to help others», and «I have kids», right? She gives a little bit of herself. And even though I understand that it’s a bad idea to become best friends with your therapist… it’s not bad. For me, I can easily separate a friend from a therapist, right? But as you can see here, I become more active, and I immediately become more interested. And I actually said that to her. «I trust you more when you tell me things about yourself, too». Right? /…/ Because, in a way, you tell *me* that «you’re (.) a person, not just a patient», or a number in line, right? /…/ Yes, I’m more interested. It changed instantly when she started talking about herself. (Elizabeth)
T: We have to tidy up and be the master of our own house first before we are able to help others (P and T talking at the same time, inaudible)	
P: Uhm. I do understand that I have to do things differently than what I’ve done before	
T: That applies to me too. My job is to help other people, really, or try to help other people. And I have children, of course, that I need to help, too	
P: Yes	
T: But I’m bound to look after myself in the middle of all of this, too. Or it will not end well	
Theme I «T providing reciprocity and intonation; P feeling close and trusting; P opening up» (Martha)	
Context: P and T touching on P’s trauma narrative when they work on maladaptive thoughts	P: That’s (therapist’s name). /…/ She was able to do that. /…/There was something about her and me. I found something in her that was so *true*. /…/ There was a connection between her and me. I can’t explain what it was. But I trusted and *trust* her completely. She made me hear and listen, and I *believed* her. /…/ There was something with her that made, her whole *being* in a way, she made me understand that she believed me, she listened to me, and it was like, after ten sessions she suddenly merged three words from another session. So, she was genuinely *interested* in me. /…/ And we had a few of those, we had many *good* moments together. I’m not sure that everyone get to experience that. But she was… she was (therapists’ name) (laughing). I’ve tried to figure out [if she reminded me of someone], almost like «what is it that she has that no one else has?». I shouldn’t say that no one else has that, but she, she came through for me and touched me. /…/ No, but I think that we (hesitating), I choose to believe that we were a very good match, that she was genuinely interested and (hesitating)… She was very much inside my mind, I mean, at times even more on my inside than she was supposed to, inside of what I was telling her. And I guess that made me feel that she really meant it. Yes, she *meant* it. It wasn’t like «that’s it for today». /…/ And that’s… yeah, that’s the difference between a therapist and a *therapist*. (Martha)
P: Locked me out… when they’d locked me up, wondered where I was, but they always found me again. My God. There were no child protective services back in those days. Damn it	
T: (inaudible)	
T: For sure. I haven’t felt that. I mean, I I’ve been angry at my mum and dad but (heavy breathing, hand on chest) wow, wow… wow	
T: I hope and believe that a few of the reasons that (inaudible), is that we have challenged a few of those thoughts. Because if you accept that «I’m the one who’s not good enough», then	
P: (interrupting) That’s what I’ve done over time, although I in a way… I haven’t done that, but at the same time I have, do you get that? Because I’ve tried so hard to become the opposite. And then it came to all of that (pointing at text on whiteboard)	
T: Yes, because that’s what happens very often when one has the feeling that «I’m never good enough» (inaudible) that you’re supposed to push yourself all the time, so that you can be good enough for someone. Or be *important* enough for someone. Significant enough. And that can also be a good thing, not that it’s necessarily	
P: (interrupting, laughing) is crazy (sighing) Yes	
T: So that became a lot to…	
P: (drying tears) I guess it did. Yes, it did. (sighing). It really did	
Master theme III: P joining T’s verbal, reflective interventions and detaching from T’s experiential techniques during trauma work (Elizabeth, Sandra, Martha, Maria)	
Theme J «P becoming overwhelmed during associative trauma-related processing; P emotionally unavailable to T» (Elizabeth, Sandra, Martha, Maria)	
Context: P and T going through P’s reactions during and following trauma exposure	P: Uhm, I’ve had a few of those. It’s like a hole, like I said to begin with, that you sort of walk down in a, uhm, deep, deep, deep down, everything becomes dark, nothing means anything. Nothing, uhm. /…/ No, I don’t think [I’m scared when that happens] /…/ When that happens, everything turns dark. But scared? No, I don’t think so. /…/ I think I had some of the same, or, was experiencing the same feeling, but I was not, I wasn’t depressed, if you get what I mean, I just experienced some of the same. /…/ I don’t have much contact with my body, really. /…/ I guess I get what I need, yes. /…/ It was really like, to talk about it, and in a way put things in order a little bit. (Maria)
P: It’s like the feeling that you just, like capitulate, so it’s very strong feelings (inaudible) it didn’t mean anything, and all the rules I have about food, none of it meant anything	
T: And to think about the difficulties, and, what you’ve been through during childhood, right, and still experience	
P: Yes, like that. And right there and then, everything became dark. It was like something that (crying). But there’s so much feelings in it	
T: And did tears come, or was there anything?	
P: Yes, I cried. I couldn’t hold anything back. I was a bit stressed and I just (crying), uhm	
T: And was it the same?	
P: (interrupting) was the same thing, mm (crying). It was	
T: It’s good that it’s better now, what do you think have contributed to that during the weekend?	
P: Things went a bit better, uhm like, it was Wednesday, and Fri-, no I guess Thursday was a bit better, I think/…/ And then it just settled in a way. It’s incredibly painful to experience that	
Theme K «T and P reflecting on P’s post-trauma responses; P staying with the topic; P increasing understanding of self» (Martha, Maria)	
Context: T and P exploring how childhood trauma may affect self-worth	P: I really (stuttering), you know that’s how it’s been, in a way. Logically. But it feels good to go through things step-by-step and I really felt that it felt very good to like… I felt good about many things that we did. Not just in this session, but generally to get things into order, as I would call it. Things that have felt illogical, or things that have been said to me. And then I get to hear «that, that, and that… equals this». And it felt so good to get some of my experiences confirmed, for instance, with physical abuse and that, and where I’ve ended up today. It feels very good to frame it. Do you understand what I mean? /…/ Being affirmed. And «this was not right», and also «when you’ve experienced that, this may happen» right? And I’ve been thinking a lot about this from time to time. But when it is not validated… Because all confirmation you’ve ever had is total opposite. Yes, you do know it, of course, but the body doesn’t want to. The feeling and (stuttering) (.) Really, the whole body is protecting itself (Martha)
T: Yes, and when that happens it’s difficult to establish a sense of «I’m good enough». Because that’s a way of showing that you aren’t good enough, «they hit me because I’m not good enough»	
P: Yes. They hit me for anything, really. If I had out my shoes on the wrong way /…/ We’re talking about things that you wouldn’t logically punish a child for doing. No. I believe it has nothing at all to do with that. Yes	
T: (inaudible) not just your actions, but also you being wrong	
P: That’s how I felt. Because no matter what I did or didn’t do, it was all wrong. What I did wrong yesterday could for instance be that if I put my shoes to the left, I did what my mother told me to do and put them to the right. And then, when I put them to the right the next day it was wrong, I should’ve put them to the left. Really, there were always, I never knew (stuttering) what was the rule. Because there were no rules, the rules kept changing./…/ The changed according to what felt good to her, and then, she needed someone to scream at, yell at, and hit, and that was me. It was like, that was not the exception, but rather, the rule	
Theme (diverging case) «T deepening and containing P’s emotional experiences in a composed manner; P opening up and exploring; P increased trust and stronger bond with T» (Alice)	
Context: P and T exploring P’s relationship to her mother	P: Uhm, I remember that it became a bit chaotic because I didn’t, I’m not that used to crying that much, and especially not in front of people, but she got something out of me that (.) meant something for our shared relationship in therapy, she really showed me that she understood what’s the issue, and *how* that’s an issue. It definitely did something to our relationship. It became, it really came to that I worked more with what she, uhm, she tried to evoke (.) at least during that time, but I don’t know, being listened to, understood, getting in contact with my tears, and she’s still composed and (laughing) meeting my eyes when I look at her and, yes, available and helpful even though I started crying. (Alice)
P: Yes, she, every time we talk about that stuff, she always becomes uhm upset /…/ Guilt, really. And we have talked a lot about it, uhm, and yeah, she feels a lot of guilt about not being there for me, or that she has more than enough of her own stuff to struggle with, and then she goes	
T: (interrupting) and perhaps there is shame there, too?	
P: Yeah, I think so. She had me when /…/, she was very young	
T: Mm. Yes. But she ends up embracing you, listening to how it was for you, feeling the pain, she could’ve well have reacted in another way, not having the courage to do it, feeling too ashamed, that she couldn’t deal with, not wanting to know, or rejecting you, but she doesn’t	
P: No. No, she’s the best (laughing) one could have	
T: Can you manage to get hold of some feelings inside of you when you think of your mother? «She’s the best» you say, and then there are tears. Can you feel something in your stomach?	
P: Yes, I’m just so happy that I have her	
T: Can you feel any warmth in your stomach? That’s often a sensation of joy (.) a warm sensation in the stomach	

T, therapist; P, patient; I, interviewer; (.),short pause; /…/, text omitted for communication purposes; [], information inserted to increase readability

### Overarching themes

Eleven themes (A–I) were constructed from interpretations of data and abstracted into three Master Themes (MTs, Table 4). Notably, one informant (Alice) diverged from the others’ accounts; hence, her theme is not included in the cross-case analysis (see Tables 3, 5).

**Table 4:** Themes and master themes across cases

Master theme I: P managing interactions with T through self-effacing and submissive behaviours
(A) P feeling rebuked by T; P non-assertive to avoid verbal abuse; P becoming submissive
(B) P fearing to fail therapy and upset T; P other-orienting to find ‘correct’ answers
(C) P feeling violated by T; P contempting T but pretending to participate; P increased self-contempt
(D) P submitting to T to gain approval
(E) P attempting to self-assert; P fears hurting T with increased guilt; P withdrawing assertive attempts
(F) P experiencing T as too forceful; P becoming overwhelmed and passive
Master Theme II: P preferring either closeness or distance to T to be able to open up
(G) P distancing from T to be able to open up about affect-laden, trauma-related topics
(H) T self-disclosing; P feeling closeness/blurred professional boundaries; P more interested and opening up
(I) T providing reciprocity and intonation; P feeling close and trusting; P opening up
Master theme III: P joining T’s verbal, reflective interventions and detaching from T’s experiential techniques during trauma work
(J) P becoming overwhelmed during associative trauma-related processing; P emotionally unavailable to T
(K) T and P reflecting on P’s post-trauma responses; P staying with the topic; P increasing understanding of self

**Table 5:** Individual patterns of themes

Alias	Themes
Elizabeth	A. P feeling rebuked by T; P non-assertive to avoid verbal abuse; P becoming submissive
	D. P submitting to T to gain approval
	F. P experiencing T as too forceful; P becoming overwhelmed and passive
	H. T self-disclosing: P feeling closeness/blurred professional boundaries; P more interested and opening up
	J. P becoming overwhelmed during associative trauma-related processing; P emotionally unavailable to T
Sandra	B. P fearing to fail therapy and upset T; P other-orienting to find ‘correct’ answers
	H. T self-disclosing; P feeling closeness/blurred professional boundaries; P more interested and opening up
	J. P becoming overwhelmed during associative trauma-related processing; P emotionally unavailable to T
Helena	C. P feeling violated by T; P contempting T but pretending to participate; P increased self-contempt
	D. P submitting to T to gain approval
	E. P attempting to self-assert; P fears hurting T with increased guilt; P withdrawing assertive attempts
Martha	I. T providing reciprocity and intonation; P feeling close and trusting; P opening up
	J. P becoming overwhelmed during associative trauma-related processing; P emotionally unavailable to T
	K. T and P reflecting on P’s post-trauma responses; P staying with the topic; P increasing understanding of self
Alice	T deepening and containing P’s emotional experiences in a composed manner; P opening up and exploring; P increased trust and stronger bond with T
Maria	G. P distancing from T to be able to open up about affect-laden, trauma-related topics
	J. P becoming overwhelmed during associative trauma-related processing; P emotionally unavailable to T
	K. T and P reflecting on P’s post-trauma responses; P staying with the topic; P increasing understanding of self

#### MT1: P managing interactions with T through self-effacing and submissive behaviours

MT1 comprises six themes (A–F) on patients’ self-effacing and submissive interactions with the therapist (Elizabeth, Sandra, and Helena). Patient responses to therapist behaviours perceived as criticising, violating, or forceful were submission, passivity, pretending to participate, and withdrawing self-assertion, from fearing negative consequences. Elizabeth explains that “I feel rebuked /…/ insecure about whether or not to speak my opinion/…/ scared of getting hit verbally” (A), and Helena states that “I say that clearly there and then, but then I laugh /…/ because I don’t want to hurt her feelings” (E). Other salient themes concerned other-orienting towards the therapist’s presumed agenda due to a fear of failing therapy, as Sandra states, “I know what to answer, but I don’t dare saying it /…/ the constant fear of saying something wrong” (B) or submitting for approval as Helena states “I’m actually sitting there lying/…/ trying to please her /…/ be good, and say what I’m supposed to” (D).

#### MT2: P preferring either closeness or distance to T to be able to open up

MT2 comprises three themes (G–I) centering on patients’ preferences of closeness or distance to the therapist to be able to open up, represented by Elizabeth, Sandra, Martha, and Maria. The two closeness-seeking strategies concerned patients’ positive responses to therapist self-disclosure that was perceived as blurring patient-therapist boundaries (H) and the positive impact of the therapist’s reciprocity and intonation regarding increased trust and opening up (I). The former was expressed by Elizabeth: “even though I understand that it’s a bad idea to become best friends with your therapist, it is not bad /…/ I’m more interested, it changed instantly when she started talking about herself” (H). The latter was exemplified by Martha, who stated that her therapist was «even more on my inside than she was supposed to at times too, inside of what I was telling her, and I guess that made me feel that she really meant it” and that she “trusted and *trust* her completely” (I). Maria, on the contrary, preferred a distanced patient-therapist relationship, describing it as “so much more difficult being open to other people” and the therapist as “a person that I, don’t have to adhere to in my daily life” *(G).*

#### MT3: P joining T’s reflective interventions and detaching from T’s experiential techniques during trauma work

MT3 comprises two themes (J–K) on patients’ access to different experiential levels, as they seemed more inclined to engage in reflective interventions than those of emotion or experience (Elizabeth, Sandra, Martha, Maria). First, patients expressed emotional detachment when overwhelmed by interventions; for example, Maria described that “everything becomes dark” and not having “much contact with the body” during experiential trauma interventions, while at the same time finding it beneficial to talk about post-traumatic reactions (J). Second, informants expressed a need for the therapist to help create logic between past and current experiences. Martha, for instance, explained how it “feels good to go through things step-by-step /…/ to hear “that, that, and that… equals this”./…/ you do *know* it, of course, yes, but the body doesn’t want to, the feeling and, really, the whole body is protecting itself” (K).

### Diverging theme

#### T deepening and containing P’s emotional experiences in a composed manner; P opening up and exploring; P increased trust and stronger bond with T

One informant (Alice) came forth with a differing narrative as her accounts included emotional exploration and expression that she perceived the therapist to contain, which in turn strengthened their relationship and provided a stronger base for exploring. She described that her therapist “got something out of me that meant something for the relationship that we share /…/ showed me that she understood, what, what’s the issue, and *how* that’s an issue” and that “it came to that I worked more with what she, uhm, she tried to evoke”. She also added that she felt “listened to, understood, getting in contact with my tears, and she’s still composed and meeting my eyes when I look at her and, yes, available and helpful even though I started crying”. This narrative contrasts the others since Alice describes agency (cf. self-effacement) and emotional experiencing (cf. avoidance) under therapist guidance.

## Discussion

This study provides insights into how six non-recovered inpatients with EDs and comorbid CT late effects experienced in-session therapeutic processes post-treatment. The Core Master Theme encompassed covert strategies of self-effacement (three informants), preferring either therapist closeness or distance to open up (four informants) and more easily adhering to reflective than experiential interventions (four informants). MTs occurred across both treatment models and were central for five informants, whereas Alice’s descriptions diverged from the general cross-case pattern. Since IPA is interpretive in nature and meant to capture idiographic nuances of the accounts, themes were at times represented by one informant only (Tables 4, 5).

The first MT, P managing interactions with T through self-effacement and submission, entailed passive, submissive patient responses (suppressing anger/contempt, withdrawing assertion) to therapist behaviours that were perceived as violating or forceful. They also included patients’ other orientation that sprung from a need for therapist approval or avoiding failure. Since patients pretended to participate, strategies seemed to occur outside therapists’ awareness: at least they were not addressed explicitly in session. Findings converged with real-time self-reported interpersonal problems pre-treatment, with general tendencies towards the non-agentic IIP-64 pole (Fig. 1), and with mid-range level treatment processes that include patients’ focus on others’ needs [28]. Findings also align with patients’ proneness to deferential acts, e.g., fearing to criticise the therapist and eagerness to fulfil therapist expectations, and responses based on what they feel they can disclose safely [31]. Alice diverged from this pattern as self-effacement was absent in her descriptions, which overlapped with her pre-treatment IIP-64 scores showing no caseness for interpersonal problems (Fig. 1).

The second MT reflecting patients’ preferences of either therapist closeness or distance to open up included two themes that referred to (a) therapist self-disclosure and (b) therapist reciprocity and intonation, with closeness and blurred patient–therapist relationships as prerequisites for opening up (Elizabeth, Sandra, Martha). On the contrary, Maria preferred distance from her therapist due to difficulties being open when feeling too close. Notably, Maria scored > 5 for WAI emotional bond during treatment week five, with a steep decline the following week and persisting until discharge, whereas Elizabeth reported low scores with a slight increase throughout. Martha and Sandra instead reported high WAI emotional bond scores during the treatment course (Additional file 4: Fig. S3). This indicates that patients’ preferences for either pole on the closeness/distance continuum in our study largely, but not unanimously, concurred with patients’ self-reported real-time emotional bonds with their therapists, as measured by WAI. Also, since all informants had poor long-term outcomes and some had high WAI emotional bond scores which would normally relate to good outcomes, it raises new hypotheses on the ED alliance-outcome association. One explanation for our findings may be that the emotional bond functions differently regarding a treatment’s goals and tasks, aligning with research showing that different types of emotional bonds may function differently in different treatment models in producing favourable outcomes [69, 70].

Findings align with traumatically attached people being more inclined to experience internal struggles between the drive to connect and the drive to fight or flee, therefore presenting with either dismissing or suspicious preoccupied tendencies with fear of abandonment or rejection [71]. We thus suggest that MT2 processes relate to differentiation dysfunction, where healthy differentiation entails parallel developmental processes of proximity seeking and increased autonomy. The ultimate goal of these parallel processes is psychological security [72 cited in 73], meaning the “freedom to explore the inner and outer world” (p. 859), i.e., not having to compensate for relational difficulties. Insecure attachment developing in childhood instead creates obstacles to healthy differentiation, leading to an overemphasis on the development of either closeness-seeking or distancing, in line with MT2 descriptions.

The third MT revealed that patients more readily engaged with reflective verbal interventions and detached from experiential techniques during trauma work. We suggest that this relates to emotion intolerance and dysregulation that may follow traumatic attachment through patients’ under-/overactivity of the stress response system. A resilient nervous system that recovers easily from distress (sympathetic activation) or boredom (parasympathetic activation) is built from consistent interactive regulation in early attachment relations, with development of self-regulation over time. When attachment figures engage in interactions that alarm the child (acting frightening/frightened), a flexible window of affect tolerance fails to develop [71]. In agreement, disorganised attachment significantly predicts dissociation, Borderline Personality Disorder (BPD), and Dissociative Identity Disorder (DID) [74], which also aligns with the pervasiveness of EDs with late CT effects resembling CPTSD [17], and the fact that dissociation seems more common in EDs with CT than in the general population and other disorders [75]. Alice who diverged from this general pattern instead described an ability to focus on the exploration and deepening of emotions, while being contained and supported by her therapist. The combination of emotional arousal and reflective exploration is generally associated with favourable outcomes across disorders and treatments [74], but did not, however, seem to be sufficient for full recovery according to established outcome criteria ([58]; Table 1).

We suggest that differentiation issues, self-effacement and emotion dysregulation interplay unfavourably in treatment; our clinically diverging case adds information to the general pattern and raises further questions. Notably, Alice differed from the others since she showed neither pre-treatment caseness for PTSD (PSS-SR) nor interpersonal problems (IIP-64), and she did not fulfil an ED diagnosis at 1-year follow-up. This suggests that she was approaching ED recovery, although self-reported residual restraint made her disqualify as partially recovered since only cognitive residual symptoms are allowed for partial recovery ([58]; Table 1). Findings underscore that CT in itself does not equal post-traumatic sequelae, but that mediators such as PTSD, emotion dysregulation, and/or interpersonal difficulties may be at play to complicate and prolong the treatment course. This concurs with research suggesting that PTSD, emotional dysregulation, interpersonal difficulties, and avoidant personality styles mediate CT’s impact on ED development, severity, and chronicity [2, 10, 12, 13].

### Clinical implications

This study targets the intersection of relational functioning and lack of long-term change in EDs with CT, and may be immediately clinically relevant since it concerns actual real-life patient–therapist interactions. Covert preoccupation with navigating the therapeutic relationship were central in the narratives of patients with EDs and CT and pre-treatment caseness for ED, interpersonal problems, and post-trauma symptoms. Awareness around patient characteristics may therefore alert clinicians to explicitly address inter- and intrapersonal processes that are potentially impeding treatment progress. Undisclosed under/overactivated states may for instance have important consequences for trauma exposure treatment since therapists risk that patients engage in covert experiential avoidance and end up without treatment effect.

### Study limitations and future research

This study has some limitations. First, informants were purposefully selected based on outcome assessments at 1-year follow-up, and since we were not blinded to outcomes, this may have coloured our interpretations to emphasise unfavourable aspects of the process accounts. Second, although findings highlighted processes in a patients with poor long-term outcomes, we cannot claim these processes to *produce* poor long-term ED outcomes, and we call for caution about any causality claims. Also, the lack of control group made us unaware of whether the same processes occur also in *favourable* long-term outcomes. Third, generalisability is limited due to the small sample size, although we do recognise patients’ accounts as important in generating novel information in line with an explorative aim and methodology that do not make use of predetermined categories, and thus is suitable for complex, understudied topics. Fourth, patients’ ability to express themselves about complex processes may vary, and to help informants counteract possible biases, we used IPR to focus informants’ attention on their own mental processes and provide suitable stimuli as recommended [64]. Fifth, the less-than-ideal and varying time intervals between treatment and IPR interviews may have impacted our findings through which data was ultimately obtained. However, although interpretations of past events are subject to change over time, descriptions also seem to mature and enrich over time [77]. Moreover, the evocative nature of IPR likely helps informants come into contact with and recall experiences better than regular interviews, where time intervals may be a more crucial issue. Importantly, we prioritized travelling to the informants if they wished to be interviewed at home, and thus, we compromised this time interval to capture rich data from these often poorly functioning patients, who may not have been able to participate in the study otherwise. Sixth, informants’ interpersonal styles, such as self-reported non-agentic tendencies (non-assertive, overly accommodating, socially inhibited), may have played out during the interviews. This potential source of error may, however, have been partly counteracted by the interviewer being a licenced psychologist with clinical experience.

Accordingly, we propose that future research explore moment-to-moment interactions for good outcomes to disentangle processes associated with a successful treatment course. Also, by continuing sampling and through other analytical methods focusing on theory building [68], it is possible to construct contextualized, explanatory models. In order to make inferences, larger samples and repeated measures designs could test models within and across treatment contexts and shed further light on how variables interrelate over time.

## Conclusion

Our findings provide novel insights in terms of plausible ED treatment modifications, as we revealed that a sample of poor outcome ED patients CT were predominantly preoccupied with calibrating the emotional–relational landscape of the patient-therapist relationship in-session; we hypothesize that differentiation dysfunction, psychological insecurity and affective intolerance limited patients’ freedom to explore own experiences. One remaining question, however, is whether working on patients’ relational difficulties has spillover effects on the ED, or whether relational issues are hindering factors that need to be eliminated for patients to benefit from more targeted ED treatments.

## Supplementary Information

**Additional file 1.** Semi-structured Interview Guide on Therapeutic Micro-processes in Inpatient ED Treatment.

**Additional file 2.** Individual WAI Tasks subscale scores during treatment.

**Additional file 3.** Individual WAI Goals subscale scores during treatment.

**Additional file 4.** Individual WAI Emotional Bond subscale scores during treatment.

## Data Availability

This study is situated within a randomized trial (Clinical trials: NCT02649114), using interview data as primary material. The data are not publicly available due to privacy or ethical restrictions, but may be available on request from the corresponding author.
