# Supplementary material for: Covert therapeutic micro-processes in non-recovered eating disorders with childhood trauma: an interpersonal process recall study
Source: J Eat Disord. 2022 Mar 21;10:42. doi: 10.1186/s40337-022-00566-1 (PMC8935733; doi:10.1186/s40337-022-00566-1)
Supplement: Supplementary file 1 — Additional file 1. Semi-structured Interview Guide on Therapeutic Micro-processes in Inpatient ED Treatment. [file 40337_2022_566_MOESM1_ESM.docx]

**Appendix 1. Semi-structured Interview Guide on Therapeutic Micro-processes in Inpatient ED Treatment.**

Introduction

This interview aims to capture your personal experience of the treatment you have recently received, with a particular focus on individual therapy processes. Your experiences are very valuable information that may help us understand the treatment of eating disorders for those who have also experienced difficult relationships and episodes during their childhood. This information may, in turn, help us to improve eating disorder treatment for this particular patient group.

To stimulate your recall and capture your inner experiences as they were at the moment, we will be viewing one of your individual therapy sessions. You are in charge of where you would like to stop the tape, and I will pose some questions as we go. I would like you to particularly look for moments that were of significance to you, and that you would like to explore further. That way, we can explore what you were experiencing there and then. If you need a break during the interview, please let me know.

Examples of prompts around therapy processes regarding the sequences to which the informants gave salience and selected:

- When X happened in the video, what were you thinking?
- What were you feeling during that moment?
- Did you notice changes in your body there and then?
- Was there anything you felt like saying there and then, but for various reasons, never said?

As the video recording reaches the end, the interviewer expresses gratitude to the informant for participating and closes the interview.
