# Supplementary figures and images for: Covert therapeutic micro-processes in non-recovered eating disorders with childhood trauma: an interpersonal process recall study
Source: J Eat Disord. 2022 Mar 21;10:42. doi: 10.1186/s40337-022-00566-1 (PMC8935733; doi:10.1186/s40337-022-00566-1)

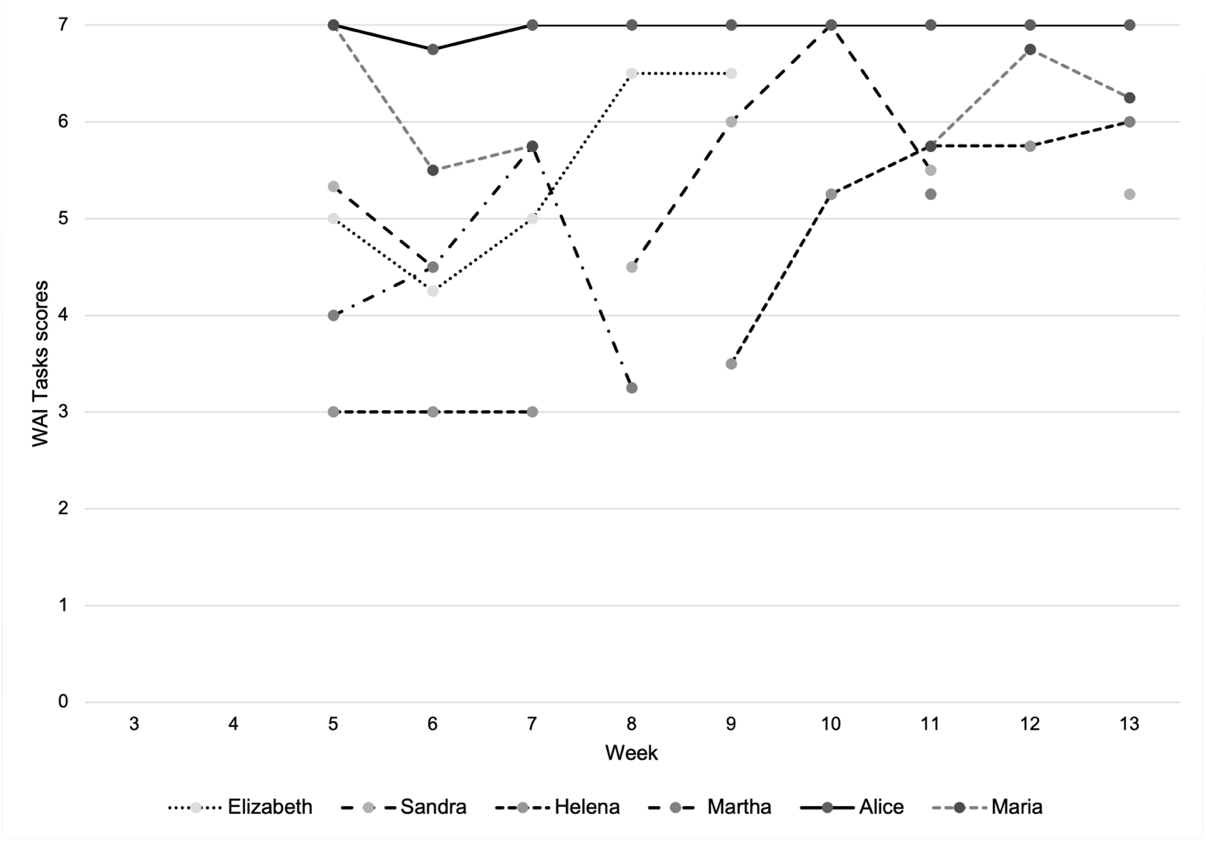


Supplemental figure 1. Individual WAI Tasks subscale scores during treatment.

Supplement: Supplementary file 2 — Additional file 2. Individual WAI Tasks subscale scores during treatment. [file 40337_2022_566_MOESM2_ESM.docx]

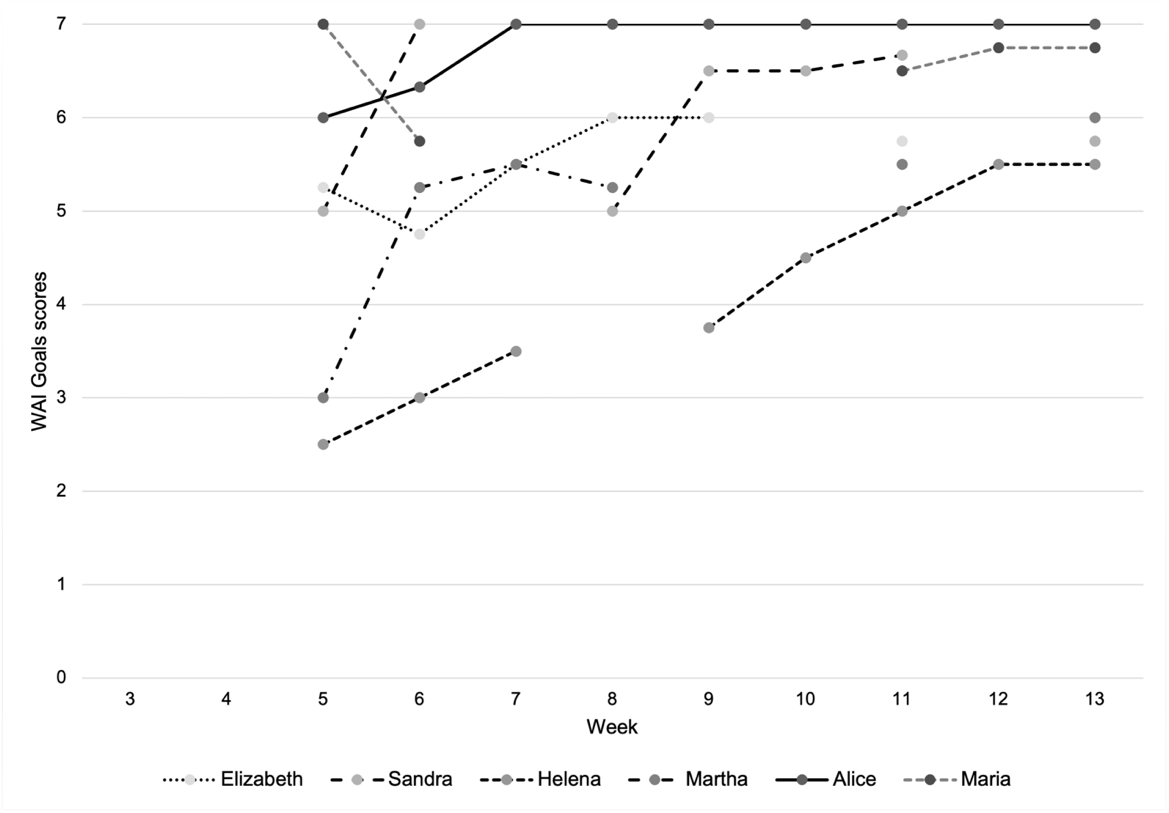


Supplemental figure 2. Individual WAI Goals subscale scores during treatment

Supplement: Supplementary file 3 — Additional file 3. Individual WAI Goals subscale scores during treatment. [file 40337_2022_566_MOESM3_ESM.docx]

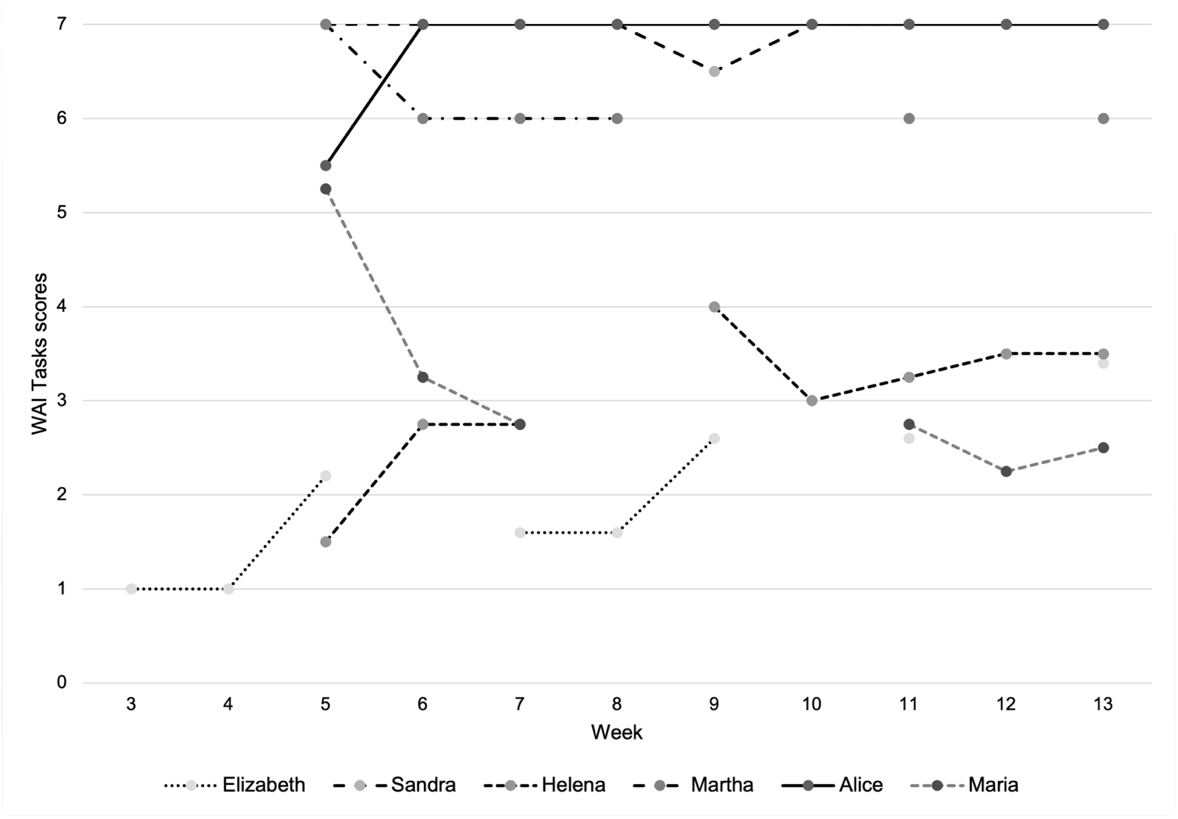


Supplemental figure 3. Individual WAI Emotional Bond subscale scores during treatment

Supplement: Supplementary file 4 — Additional file 4. Individual WAI Emotional Bond subscale scores during treatment. [file 40337_2022_566_MOESM4_ESM.docx]
